# Drought Tolerance in Modern and Wild Wheat

**DOI:** 10.1155/2013/548246

**Published:** 2013-05-15

**Authors:** Hikmet Budak, Melda Kantar, Kuaybe Yucebilgili Kurtoglu

**Affiliations:** Biological Sciences and Bioengineering Program, Faculty of Engineering and Natural Sciences, Sabanci University, 34956 Tuzla, Istanbul, Turkey

## Abstract

The genus *Triticum* includes bread (*Triticum aestivum*) and durum wheat (*Triticum durum*) and constitutes a major source for human food consumption. Drought is currently the leading threat on world's food supply, limiting crop yield, and is complicated since drought tolerance is a quantitative trait with a complex phenotype affected by the plant's developmental stage. Drought tolerance is crucial to stabilize and increase food production since domestication has limited the genetic diversity of crops including wild wheat, leading to cultivated species, adapted to artificial environments, and lost tolerance to drought stress. Improvement for drought tolerance can be achieved by the introduction of drought-grelated genes and QTLs to modern wheat cultivars. Therefore, identification of candidate molecules or loci involved in drought tolerance is necessary, which is undertaken by “omics” studies and QTL mapping. In this sense, wild counterparts of modern varieties, specifically wild emmer wheat (*T. dicoccoides*), which are highly tolerant to drought, hold a great potential. Prior to their introgression to modern wheat cultivars, drought related candidate genes are first characterized at the molecular level, and their function is confirmed via transgenic studies. After integration of the tolerance loci, specific environment targeted field trials are performed coupled with extensive analysis of morphological and physiological characteristics of developed cultivars, to assess their performance under drought conditions and their possible contributions to yield in certain regions. This paper focuses on recent advances on drought related gene/QTL identification, studies on drought related molecular pathways, and current efforts on improvement of wheat cultivars for drought tolerance.

## 1. Introduction

Current climate change is projected to have a significant impact on temperature and precipitation profiles, increasing the incidence and severity of drought. Drought is the single largest abiotic stress factor leading to reduced crop yields, so high-yielding crops even in environmentally stressful conditions are essential [1, 2]. This is not the first time we face this situation, in which increasing demands on existing resources are not feasible, and higher-yielding crops are required to balance crop production with increasing human food consumption. A similar scenario occurred 50 years ago due to the high rate of population growth, and it was overcome by selective breeding of high grain yielding semidwarf mutants of wheat, a process coined Green Revolution [3]. In relation to current development of cultivars, which are higher yielding even in water-limited environments, one of the major targets is *Triticum* species, being one of the leading human food source, accounting for more than half of total human consumption [2, 4]. 

The increasing incidence and importance of drought in relation to crop production has rendered it as a major focus of research for several decades. However, studying drought response is challenged by the complex and quantitative nature of the trait. Drought tolerance is complicated with environmental interactions. In the analysis of a plant's drought response, the mode, timing, and severity of the dehydration stress and its occurrence with other abiotic and biotic stress factors are significant [5]. Furthermore different species, subspecies, and cultivars of crops show variation in their drought tolerance under same conditions, emphasizing the importance of genetic diversity as an underlying factor of drought and its significance in drought-related research. Plants exhibiting high drought tolerance are the most suitable targets of drought-related research and are the most promising sources of drought-related gene and gene regions to be used in the improvement of modern crop varieties. These include the natural progenitors of cultivated crops, and for wheat improvement, *Ae. tauschii, *which is more drought tolerant than *Triticum* and wild emmer wheat (*T. dicoccoides*), which harbors drought tolerance characteristics, lost during cultivation of modern lines, is of great importance [6]. 

Although development of higher-yielding crops under water-limited environments is the most viable solution to stabilizing and increasing wheat production under current climatic conditions, it is challenged by the nature of drought response as a trait and the complex genomic constitution of wheat [16]. However, recently, the utilization of drought tolerant wild species and the rapid advances in molecular biological, functional genomics, and transgenics technologies have facilitated drought-related studies, resulting in significant progress in the identification of related genes and gene regions and dissection of some of its molecular aspects. This paper summarizes the current state of drought-related research in *Triticum* species, focusing on the identification and functional characterization of drought-related molecules, analysis of their interactions in the complex network of drought response, and applications of these data to improve wheat cultivars utilizing molecular based-technologies.

## 2. *T. dicoccoides *and Drought Tolerance

Wild emmer wheat (*T. turgidum *ssp.* dicoccoides* (körn.) Thell) is the tetraploid (2*n* = 4*x* = 28; genome BBAA) progenitor of both domesticated tetraploid durum wheat (*T. turgidum *ssp.* durum* (Desf.) MacKey) and hexaploid (2*n* = 6*x* = 42; BBAADD) bread wheat (*T. aestivum* L.). It is thought to have originated and diversified in the Near East Fertile Crescent region through adaptation to a spectrum of ecological conditions. It is genetically compatible with durum wheat (*T. turgidum* ssp.* durum*) and can be crossed with bread wheat (*T. aestivum* L.) [17]. Wild emmer germplasm harbors a rich allelic pool, exhibiting a high level of genetic diversity, showing correlation with environmental factors, reported by population-wide analysis of allozyme and DNA marker variations [18–24].

Wild emmer wheat is important for its high drought tolerance, and some of *T. dicoccoides* genotypes are fully fertile in arid desert environments. Wild emmer wheat accessions were shown to thrive better under water-limited conditions in terms of their productivity and stability, compared to durum wheat. The wild emmer gene pool was shown to offer a rich allelic repertoire of agronomically important traits including drought tolerance [23, 25–28]. Hence, *T. dicoccoides* is an important source of drought-related genes and highly suitable as a donor for improving drought tolerance in cultivated wheat species.

Wild emmer wheat, being a potential reservoir of drought-related research, has been the source of several identified candidate drought-related genes with the development of “omics” approaches in the recent decades. In recent years, transcript profiling of leaf and root tissues from two *T. dicoccoides* genotypes, originating from Turkey, TR39477 (tolerant variety), TTD-22 (sensitive variety), was performed by our group, in two separate studies, utilizing different methodologies. In one report, subtractive cDNA libraries were constructed from slow dehydration stressed plants, and over 13,000 ESTs were sequenced. In another study, Affymetrix GeneChip Wheat Genome Array was used to profile expression in response to shock drought stress [1, 29]. Wild emmer wheat was shown to be capable of engaging in known drought responsive mechanisms, harboring elements present in modern wheat varieties and also in other crop species. Additionally several genes or expression patterns, unique to tolerant wild emmer wheat, indicative of its distinctive ability to tolerate water deficiency, were also revealed. Transcript and metabolite profiling studies were also undertaken for two *T. dicoccoides* genotypes, originating from Israel, Y12-3 (tolerant variety) and A24-39 (sensitive variety), under drought stress and nonstress conditions. Leaf transcript profiling indicated differential multilevel regulation among cultivars and conditions [30]. Integration of root transcript and metabolite profiling data emphasized drought adaptation through regulation of energy related processes involving carbon metabolism and cell homeostasis (Table 1) [14]. Recently, in wild emmer wheat, our group also profiled drought induced expression of microRNA (miRNAs), small regulatory molecules known to be involved in several cellular processes including stress responses. In this study, leaf and root tissues of resistant wild emmer wheat varieties, TR39477 and TR38828, were screened via a microarray platform, and 13 differentially expressed miRNAs were found to be differentially expressed in response to drought (Table 1) [15]. 

Following the identification of *T. dicoccoides* drought-related gene candidates, as discussed previously, a number of these potential drought resistant genes were cloned and further characterized. In one of the recent reports, TdicTMPIT1 (integral transmembrane protein inducible by Tumor Necrosis Factor-*α*, TNF-*α*) was cloned from wild emmer root tissue and shown to be a membrane protein, associated with the drought stress response, exhibiting increased levels of expression, specifically in wild emmer wheat upon osmotic stress [31]. In a different study, TdicDRF1 (DRE binding factor 1), conserved between crop species, was cloned for the first time from wild emmer wheat. Its DNA binding domain, AP2/ERF (APETALA2/ethylene-responsive element binding factor), was shown to bind to drought responsive element (DRE), using an electrophoretic mobility shift assay (EMSA). It was revealed to exhibit cultivar and tissue specific regulation of its expression, through mechanisms involving alternative splicing [32]. Moreover, the relations between autophagy and drought response were analyzed in another line of research by the cloning of TdATG8 (autophagy related protein 8) and its further functional investigation with yeast complementation assay and virus induced gene silencing (VIGS) of plants. In this study, autophagy was shown to be induced in drought-stressed plants in an organ-specific mode, and silencing of ATG8 was shown to decrease drought tolerance of plants, revealing it as a positive regulator of drought stress [33] (Tables 2 and 3). 

## 3. Phenotyping for Drought Tolerance in Wheat with Physiological Traits

For screening out transgenic wheat lines with desirable drought tolerance, the physiological traits and processes which can be genetically manipulated to improve wheat adaptation to drought have to be taken into account. The genetic basis of drought tolerance in wheat is still elusive. At present the physiological traits (PTs) linked to heat tolerance appear to be a superlative accessible tool since they exhibit the favorable allele combination for drought tolerance. Such alleles interact with the environment and genetic background which includes variation in gene expression and hence are still poorly understood through the QTL approach [50]. Hybridization of heat tolerance PTs may not always have a predictable outcome related to net crop yield particularly in varying environmental conditions, but breeding such varieties with complementary PTs could augment the cumulative gene effect [51]. Thus the physiological phenotyping along with gene discovery can be valuable to pin down desired alleles and understand their genetic mechanism [50]. Cossani and Reynolds have proposed a model based on this concept of genetically characterized PT for improved drought tolerance of wheat [52]. The model focuses on 3 major genetic parameters of yield when water and nutrients are not limiting factors. The genetic parameters are discussed in the following.

### 3.1. Light Interception (LI) Traits

#### 3.1.1. Canopy Architecture

Since increase in temperature is linked with a decrease in green area duration and leaf area index, light interception or LI traits can be manipulated by studying the variation in the rapid ground cover (RGC) and leaf senescence of wheat. RGC shows genotypic variability in relatively heritable and simple breeding targets such as embryo and grain size, specific leaf area, or seedling emergence rate [53]. Optimized distribution of light may improve radiation use efficiency (RUE) and LI traits since wheat displays a vast diversity in canopy structure. Furthermore leaves are more erect and smaller in size in many modern cultivars thereby facilitating RUE and allowing more light penetration to lower leaves. 

#### 3.1.2. Hindrance of Leaf Senescence

Leaf senescence during drought can be hindered by delayed expression of senescence related green thereby giving stay-green (SG) genotypes with normal photosynthesis [54]. Stay green is thus identified as an important adaptive PT for drought stress conditions, but its role in improving grain yield in drought is still a matter of extensive research. However, some correlations were shown between SG and yield and identified QTLs in mapping populations [55]. Since chlorosis in plants is not expressed homogenously in plant organs aboveground, many approaches have been developed to estimate SG including spectral reflectance, but these also need to be more specific to functional SG.

### 3.2. Radiation Use Efficiency Traits

#### 3.2.1. Photosynthesis and Photorespiration

According to Cossani and Reynolds, once the LI traits are optimized the focus on increased crop biomass will depend on RUE traits which include dark respiration, photorespiration, and other photosynthetic strategies. A central player of the photosynthetic pathway, Rubisco was observed to show lower affinity for CO_2_ over O_2_ in higher temperatures [52]. Thus, increasing the affinity of Rubisco is especially significant for adaptation to warm conditions. The importance of CO_2_ fixation by Rubisco for high temperature adaptation is also emphasised by the observation that C4 plants adapt to warm conditions by concentrating CO_2_. Present transgenic attempts to convert C3 plants into C4 plants are still in progress and require more knowledge of the maintenance of the C4 pathway. Studies of the Rubisco kinetic properties of *Limonium gibertii* may be used in transgenics in wheat even though wheat Rubisco has an excellent CO_2_ affinity. One model shows 12% increase in net assimilation when substrate specificity factor of wheat Rubisco was replaced from *L. gibertii * [56]. Rubisco activase active sites become inactive progressively under drought, thus associating the activase with heat shock chaperone cpn60*β* could provide Rubisco protection [57]. This has great potential since thermotolerant types of Rubisco in tropical species and diverse optimum temperature of Rubisco have been found in nature [58]. By exploiting this fact a chimeric enzyme was created thus increasing the heat resistance in Arabidopsis by combining the Arabidopsis Rubisco recognition domain and tobacco activase [55]. 

## 4. Identification of Drought-Related Genes and QTLs

Prior to focusing on individual drought-related components, drought response, due to its complex nature, must be viewed as a whole system, for which large scale identification of probable dehydration stress-related genes or QTLs is necessary. Potential markers for stress tolerance can be identified either through “omics” studies or QTL mapping of yield related traits under drought prone environments. In the long run, these markers can aid in screening cultivars for drought tolerance/sensitivity and/or improvement of drought tolerance in wheat.

### 4.1. Drought-Related Gene Identification by “Omics”

“Omics” techniques examine all or a representative subset of an organism's genes, transcripts, proteins, or metabolites. As well as accumulating genomic sequence knowledge, data from profiling studies is also crucial in understanding the drought response, which is largely mediated by differential accumulations of drought-related components. 

In the recent decades, high-throughput profiling techniques have been utilized for the identification of potential drought tolerance markers from different wheat species (Table 1). Some of the large scale profiling studies undertaken in wild emmer wheat were mentioned in Section 2 [14, 15, 59]. “Omics” studies were also performed to monitor dehydration induced transcripts and proteins of bread and durum wheat cultivars with differing sensitivities to drought, both in stress and nonstress conditions. Methodologies used in transcript profiling studies range from cDNA microarrays to cDNA-AFLP (amplified fragment length polymorphism). For differential protein identification, the common procedures used include 2D (2-Dimensional) gels, various chromatography techniques, and mass spectrometry. In these recent high-throughput studies, molecular mechanisms behind various drought induced physiological or morphological events were targeted, using related tissues and appropriate mode/timing/severity of stress treatments for each profiling experiment. In two of these studies, underlying molecular mechanisms of early grain development upon shock dehydration response and root functional responses upon moderate drought at tillering were investigated in bread wheat by transcript profiling [8, 60]. Proteome profiling was established in several bread wheat tissues: grain upon drought at terminal spikelet or at anthesis; leaf under field like cyclic drought conditions after first flag leaf formation; stem upon progressive drought stress after anthesis were established. The latter research was conducted to understand the underlying molecular mechanisms of mobilization of stem carbohydrate reserves to grains, a process that contributes to yield under terminal drought conditions and its findings pointed out to the involvement of senescence and protection against oxidative stress in effectiveness of the mobilization process [9]. In recent years, transcript profiling in durum wheat flag leaf upon field like drought at booting was performed [12]. In a different line of research, proteomic profiles of *T. durum *mature embryos were established, which is especially important since embryos are good model systems for drought studies, sustaining germination in extreme conditions of desiccation [13]. An overview of recently established profiling studies is provided in Table 1.

### 4.2. QTL Mapping

Dissection of drought tolerance, a complex quantitative phenotype, affected by multiple loci requires the identification of related quantitative trait loci (QTLs). QTL cloning is a large effort in terms of the technology, resources, and time required, but determination of QTLs is proceeded by great advantages in applications of marker-assisted selection (MAS) and better yielding cultivar development. Identification of QTLs takes advantage of molecular maps, developed by the use of DNA markers. The establishment of these molecular maps has been enabled by the recent advances in functional genomics, which have supplied bacterial artificial chromosomes (BACs), gene sequence data, molecular marker technology, and bioinformatic tools for comparative genomics. Mapping and fine mapping for the identification of candidate regions for a trait prior to positional cloning requires suitable mapping populations: recombinant inbred lines (RILs) and near isogenic lines (NILs), several of which have been established for wheat varieties. However, up to now, only a limited number of studies has succeeded in the positional cloning of wheat QTLs and none in the context of drought [2, 4].

In recent years, several yield QTLs were identified in wheat through linkage analysis and association mapping. Since yield is the most crucial trait to breeders, most QTLs for drought tolerance in wheat have been determined through yield and yield related measurements under water-limited conditions [64–68]. However, these studies are challenged by the factors that yield and drought are both complex traits, involving multiple loci and showing genotype and environment interactions. Yield is difficult to be described accurately with respect to water use, and its accurate phenotyping is a challenge since QTLs established in one environment may not be confirmed in other. For this reason, large scale phenotyping trials, carried out in multiple fields, taking into consideration the environmental varieties are crucial. Until now, a number of studies have identified QTLs associated with specific components of drought response using *T. durum, T. aestivum,* and *T.durum *X* T. dicoccoides* mapping populations; however the genomic regions associated with individual QTLs are still very large and unsuitable for screening in breeding programmes. However, in recent years, several yield related QTLs were mapped using (*T. aestivum* L.) RAC875/Kukri doubled haploid populations grown under a variety of environmental conditions including nonirrigated environments. In one study, inbred population was assessed under heat, drought, and high yield potential conditions to identify genetic loci for grain yield, yield components, and key morphophysiological traits [69]. In another study, regions associated with QTLs for grain yield and physical grain quality were assessed under 16 field locations and year combinations in three distinct seasonal conditions [70]. In a third study, QTLs were identified for days to ear emergence and flag leaf glaucousness under southern Australian conditions [71]. Another multienvironmental analysis provided a basis for fine mapping and cloning the genes linked to a yield related QTL [72]. These recent studies are promising, and along with the recent advances in DNA sequencing technology and new approaches of coupling linkage analysis with “omics” studies, these data will find their way into practical wheat breeding programmes in relation to drought [2, 4]. Drought-related QTLs identified in these studies are listed in Supplementary Table 1 (see Supplementary Materials available online at http://dx.doi.org/10.1155/2013/548246).

## 5. Identification of Molecular Mechanisms Related to Drought

Probable drought-related genes and QTLs, identified in “omics” and “QTL mapping” studies, should be further characterized, prior to their use in the development of better yielding cultivars. Elucidation of these components includes analyzing their gene and protein structure and determining their roles and interactions in the complex network of stress response signaling. Their functional relevance to drought should be shown and eventually confirmed with transgenic studies. This section summarizes the recent research regarding the characterization of drought-related genes, in detail, dissection of drought-related molecular pathways, and functional genomics studies. 

### 5.1. Characterization of Drought-Related Genes

Prior to its utilization for drought tolerance improvement, for each putative drought-related gene region or molecule identified in “QTL mapping” or “omics” study, the immediate step is the cloning and in detail characterization of the gene and its protein. This process exploits a variety of *in silico* and basic molecular biology methods and involves several aspects that differ on the nature of the research, including analysis of gene and protein structure, phylogeny-based studies and determination of gene chromosomal localization, protein-protein and protein-DNA interactions, and transcript and protein subcellular localizations. Further characterization involves transcript and protein monitoring in response to stress conditions and functional analysis of the protein. Utilizing these strategies, in recent years several drought-related proteins were elucidated, the majority being stress-related transcription factors (TFs) and signal transducers.

**Figure 1:**
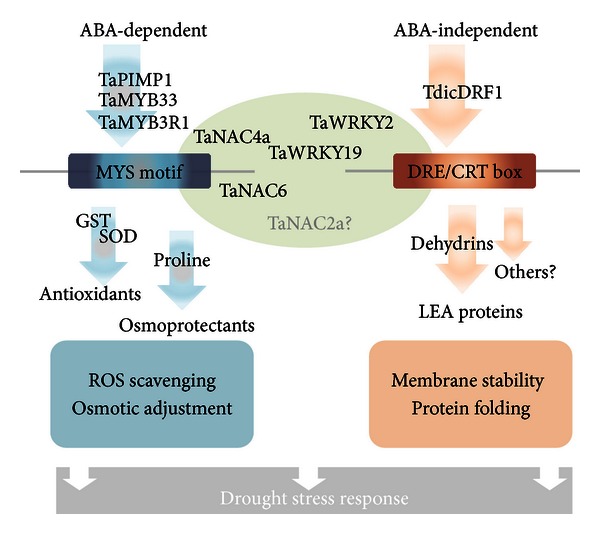
ABA-dependent and ABA-independent pathways of stress response. MYB and DREB TFs are given as examples to ABA-dependent and-independent routes. While ABA-dependent pathways appear to recruit antioxidant and osmoprotectant mechanisms, ABA-independent pathways generally involve protective proteins. NAC and WRKY TFs provide crosstalk between these pathways; where some members, such as TaNAC4 and TaNAC6, may predominantly act in an ABA-dependent fashion, some members may be closer to ABA-independent pathways. In several cases, such as TaWRKY19, both pathways are employed. It should be noted that both pathways are highly intermingled, and functions of several regulators, such as TaNAC2a, as well as entire pathways are yet to be elucidated.

Drought is known to be regulated at the transcriptional level, and TFs have been the focus of attention for the improvement of better yielding cultivars since targeting a single TF can affect several downstream-regulatory aspects of drought tolerance. Classically, two transcriptional regulatory circuits induced by drought have been studied: ABA-dependent and DREB-(dehydration-responsive element binding protein-) mediated (ABA-independent) pathways. These pathways are schematically depicted in Figure 1. One of the major classes of TFs involved in ABA-dependent stress responses is MYB TFs, and in the recent years, there has been a focus on the elucidation of bread wheat R2R3 and MYB3R type MYB TFs, known to be involved in ABA signaling of drought. In three different lines of research, drought responsive MYBs, TaPIMP1 (pathogen induced membrane protein), TaMYB33, and TaMYB3R1, were cloned and studied via the analysis of their domains, determination of their nuclear subcellular localizations, and assessment of transcriptional activation function to proteins [34, 36, 38]. Phylogenetic analysis of their protein sequences classified TaMYB3R1 as MYB3R type and the others as R2R3 type MYB TFs. The R2R3 type MYB TaPIMP1 was originally described as the first defense related MYB in wheat; however, detailed analyses indicated that TaPIMP1 is also induced by abiotic stresses, particularly drought. In addition, the induction of its expression by ABA and its inability to bind to the DRE-box element as indicated by EMSA suggest that TaPIMP1 acts in the ABA-dependent pathways of drought response [73]. Similarly, TaMYB33, another drought responsive R2R3 type MYB, was shown to be induced by ABA treatment, and the overexpression in *Arabidopsis *plants could not detect a significant increase in DREB2, suggesting that TaMYB33 is also involved in ABA-dependent mechanisms [38]. Both TaPIMP1 and TaMYB33 appear to enhance drought tolerance through ROS detoxification and reinforcement of osmotic balance. An elevated level of proline or proline synthesis common to both TaPIMP1 and TaMYB33 mediated response is noteworthy [34, 38]. MYB3R type MYB TFs are less pronounced class of MYB proteins in stress response. TaMYB3R1, one of the few examples of MYB3R type MYBs in wheat, has been implicated in drought stress response and is also responsive to ABA, similar to TaPIMP1 and TaMYB33. However, the downstream events of TaMYB3R1-mediated drought stress response remains to be elucidated [36]. 

ABA-independent also called DREB-mediated pathways are largely governed by dehydration-responsive element-binding (DREB)/C-repeat-binding (CBF) proteins which recognize dehydration-responsive element (DRE)/C-repeat (CRT) motifs through a conserved AP2 domain. While DREB1 TFs are mainly responsive against cold stress, DREB2 TFs are more pronounced in drought stress response; although functional overlaps are possible where a certain DREB responds to multiple stresses [74]. It should be noted that DREB-mediated stress responses may also cooperate or overlap with ABA-dependent stress responses (Figure 1). A number of DREB homologs have deen identified in wheat and, although DREB2-mediated drought response is not fully elucidated yet, enhanced drought tolerance through DREB-mediated pathways is considered to involve LEA proteins [75]. As noted in Section 2, recently, a DREB2 homolog, TdicDRF1, was identified, cloned, and characterized for the first time in wild emmer wheat. Comparison of drought-stressed resistant and sensitive genotypes revealing differential expressions of TdicDRF1 suggested not only a conserved role on drought stress response but also a promising mechanism that can be utilized in improvement of wheat cultivars for drought tolerance [32]. 

In recent years, evidence has accumulated that there is crosstalk between classical ABA-dependent and ABA-independent pathways (Figure 1). The best known example of such occurrence is NAC TFs, which regulate drought stress response through both ABA-dependent and ABA-independent pathways. In a recent study, *T. aestivum* NAC (NAM/ATAF/CUC) TFs were identified *in silico*, phylogenetically classified and characterized, and their expression profiles were monitored in response to ABA and drought stress. In response to these treatments, TaNAC4a and TaNAC6 exhibited similar expression trends, suggesting an ABA-dependent regulation of drought, while in the case of TaNTL5 and TaNAC2a, the changes in the expression were not parallel [37]. In another study, WRKY type transcription factors (TaWRKY2 and TaWRKY19), which are known to be involved in plant abiotic stress response and ABA signaling were identified computationally, localized to the nucleus and shown to bind specifically to cis-element, W box. This report revealed that WRKY19 as a component of both ABA and DREB pathways, showing WRKY19 expression level, was responsive to ABA application, and in transgenic WRKY19 deficient plants, the expression levels of DREB pathway components were altered [39].

In addition to these known players of ABA-dependent and DREB-mediated pathways, other novel TFs are also discovered, and one such TF is the recently discovered *T. aestivum* salt response gene TF (TaSRG) which was shown to be induced in response to drought and ABA [35]. 

Other major targets of recent research have been enzymes that aid in reversible phosphorylation of signaling molecules in drought-related network of protein interactions. Although the major classes of stress-related kinases and phosphatases taking part in these cascades are known, namely, mitogen activated protein kinases (MAPKs), SNF-1-like kinases (SnRKs), calcium-dependent protein kinases (CDPKs), and MAP kinase phosphatases (MKPs), information regarding to these components is far from complete. For this purpose, known components should be further investigated since their exact positions and interactions in the complex signaling network are currently unknown. This has been applied recently, in two separate studies, in which SNF-1-like kinases, namely, TaSnRK2.4, TaSnRK2.7 from bread wheat, were characterized further in detail [41, 42]. In a different research, a MAP kinase phosphatase, TdTMKP1 was cloned from durum wheat, and its specific interaction with two MAPKs, TdTMPK3, and TdTMPK6, was verified, in accordance of its role as a negative regulator of MAPKs [43]. In an independent line of research, a priorly poorly characterized kinase TaABC1 (*T. aestivum* L. protein kinase) was investigated and shown to be involved in drought [40].

Although transcription factors and signal transducers have been the major focus of research in terms of characterization, in recent years other putative drought-related molecules were also isolated from wheat varieties, investigated, and supporting evidence for their roles in drought stress was obtained [31–33, 44–48]. Information regarding these studies is listed in Table 2. Most of the drought-related genes identified were confirmed either by using overexpressor plants or wheat deletion lines or silencing the gene of interest via virus induced gene silencing revealing its function [33, 39, 63] (Table 3). 

### 5.2. Studies of Drought-Related Pathways

Plants in environments prone to drought stress have developed several tolerance strategies, resistance and avoidance mechanisms, which enable them to survive and reproduce under conditions of water scarcity.

The fundamental plant drought responses include growth limitation, changes in gene expression, altered hormonal levels, induced and suppressed signaling pathways, accumulation of compatible solutes and osmoprotectant proteins, suppression of metabolism, increased lipid peroxidation with higher levels of ROS, and counter-acting increased levels of antioxidant activity. Drought is regulated both at the transcriptional level and posttranscriptional level, the latter including the action of miRNAs and posttranslational modifications for proteosomal degradation [76] (Supplementary Table 2).

#### 5.2.1. Compatible Solutes

Compatible solutes are nontoxic molecules that accumulate in the cytoplasm upon drought stress. Common compatible solutes are sugars, sugar alcohols, glycine betaine, amino acids, and proline. They are known to be involved in osmotic adjustment, function as ROS scavengers, protect proteins and cell structures, and exhibit adaptive value in metabolic pathways. In a recent study, compatible solutes in *T. aestivum* leaves were screened in response to water deficit at the reproduction stage. Major contributors to osmotic adjustment were revealed to be K^+^ in the early stages of stress and molecules including glycinebetaine, proline, and glucose, in the late stress [77]. Recently, compatible solutes were also assessed in *T. aestivum* cultivars under different irrigation regimes. Drought application decreased the levels of inorganic solutes but increased the levels of organic solutes [78]. 

#### 5.2.2. Protective Proteins

Protective proteins known to be involved in drought stress response include LEA proteins, aquaporins, heat shock proteins, and ion channels. Recently, in *T. aestivum* cultivars, changes in the transcript and protein levels of dehydrins and LEA proteins, in response to progressive drought applied at early vegetation and during its recovery, were monitored [79]. In a different study, two aquaporins were overexpressed providing direct evidence of their drought-related functions [48]. Additionally in another research, SNP and InDEL (insertion/deletion) repertoire of a Na^+^/K^+^ transporter, HKT-1, was assessed and observed to be compromised of mostly missense mutations, predominantly present in the tolerant wheat variety [80].

#### 5.2.3. Signaling

Drought signaling includes signal perception and transduction. Known drought-related signal transducers and some recent reports on their characterization and functional assessment are summarized in Sections 5.1–5.3 [40–42]. Some of these studies were performed on calcium dependent protein kinases (CDPK), which sense and respond to Ca^2+^ an important secondary messenger of signal transduction cascades. In a recent study, two bread wheat CDPKs, CPK7 and CPK12, were revealed to be molecularly evolved through gene duplication followed by functional diversification. In this study, they were shown to contain different putative cis-element combinations in their promoters, and two CDPKs were shown to respond differently to drought, PEG, salt (NaCl), cold, hydrogen peroxide (H_2_O_2_), and ABA applications [81]. Other suggested signaling molecules of drought stress network are SA (salicylic acid) and NO (nitric oxide). In recent studies, NO was shown to be present in higher levels in drought tolerant wheat variety and may have a possible role in the drought induced limitation in root growth [82]. Recently, SA was shown to aid drought tolerance through increasing accumulation of solutes [78].

#### 5.2.4. Photosynthesis and Respiration

Upon dehydration conditions, basal metabolic activities, including photosynthesis and respiration, are known to be altered in plants. Recently, *T. aestivum* and *T. durum* genotypes with differing sensitivities to osmotic stress were evaluated in terms of their gas exchange in response dehydration. Photosynthesis was analyzed in depth, in relation to ROS levels and physiological parameters, for elucidation of the mechanisms by which the tolerant cultivar sustains a high performance of water-use efficiency, maintaining respiration rate and photosynthesis even under stress conditions [83]. PS II (Photosystem II) is a key protein pigment complex of photosynthesis, which aids in light harvesting. Upon dehydration, PS II repair cycle is impaired affecting oxygen evolving process of PS II reaction center, leading to photooxidative damage. PS II has a biphasic primary phytochemistry kinetics, which is referred to as PS II heterogeneity. In recent studies, photosynthetic efficiency of PSII complex was measured in different wheat varieties under different environmental conditions and the extent and nature of this heterogeneity was assessed in detail, in relation to osmotic stress [84].

#### 5.2.5. Growth

Stress response of plants in relation to growth differentiates based both on the tissue and the severity, timing, and mode of stress applied. The degree of osmotic stress induced limitation of plant organ growth also differs from cultivar to cultivar and does not have a correlation with drought tolerance [82, 85, 86]. Recently, dehydration induced retardation of root and leaf growth was studied in detail, in relation to several components of the drought response pathway. In some of these studies, cell wall-bound peroxidases, ROS and NO, were shown to be unfavorable for root expansion and suggested to have possible roles in retarding root cell wall extension [85]. Another report analyzed the underlying mechanisms of *T. aestivum* root elongation in detail, showing that even during plasmolysis upon stress, although root elongation is retarded, new root hair cell formation is sustained [87]. In a different study, monitoring of *T. durum* leaves for ABA, water status, and leaf elongation rates upon drought stress and recovery revealed a retardation in leaf elongation even after stress recovery, suggesting that a rapid accumulation of ABA during stress may have caused the loss of cell wall extensibility [86]. 

Additionally, recently, expansin, known to be involved in cell wall loosening, was overexpressed in tobacco, confirming its role in plant water retention ability and osmotic potential [83].

#### 5.2.6. Transcriptional Regulation and Posttranscriptional/Translational Modifications

The expression of several drought-related gene products is regulated at the transcriptional level. Known drought-related TFs and some recent reports on their characterization and functional assessment are summarized in Sections 5.1–5.3 [32, 34–39]. Additional studies undertaken in the recent years include preliminary research, drought induced expression profiling of MYB transcripts, revealing TaMYBsdu1 as a potential drought-related TF and characterization of SNP and INDEL repertoire of *T. durum* DREB1 and WRKY1 [80, 88]. 

As well as on the genomic level, drought stress is also regulated at the posttranscriptional and posttranslational levels. The major players of posttranscriptional regulation are miRNAs, which have been identified in a variety of crops, including *Triticeae* species, using both computational and experimental approaches, and their expression profiling was performed in wild emmer wheat and crop model species including *Brachypodium *[15, 76, 89–92]. Posttranslational modifications include protein degradation, mostly via ubiquitination. In a recent study, the leaves of resistant *T. aestivum* cultivar were shown to exhibit a relatively small increase in cysteine protease function upon dehydration, limiting protein loss of this cultivar. In this study, a cysteine protease present only under drought conditions was detected [93]. In a different report, the functional role of a cysteine protease protein was confirmed by its overexpression in *Arabidopsis *[45].

#### 5.2.7. ROS and Antioxidants

Upon drought stress, ROS generation occurs mainly in the chloroplast and mitochondria, which results in oxidative damage and lipid peroxidation. Nonenzymatic and enzymatic antioxidants are produced by the plant to detoxify ROS. In a recent report, drought induced ROS generation and antioxidant activity was screened comparatively in the root whole cells and mitochondria of drought acclimated and nonacclimated *T. aestivum* seedlings. A special role of mitochondrial scavenging mechanisms was highlighted by the finding that in specifically nonacclimated seedlings, mitochondrial antioxidants were found to be predominantly active. In the same study, a quick increase in antioxidant mechanisms was observed with stress recovery, thus it was proposed that accumulation of high levels of H_2_O_2_ upon stress can inhibit antioxidant mechanisms [94]. Another study was undertaken in two *T. aestivum* cultivars, differing in their drought tolerance, focusing especially on the level and activity of ascorbate glutathione cycle related enzymes in stem and leaf tissues. It was shown that ascorbate processing and oxidation were differentially changed in the two cultivars [7]. Recently several other studies of ROS were performed to determine its role in the drought response network in relation to photosynthesis, NO, root growth, ABA, and BABA (*β*-aminobutyric acid) [82, 95, 96].

#### 5.2.8. Abscisic Acid

ABA is a plant hormone that is known to be involved in plant developmental processes and was also shown to be an inducer of stress-related pathways. Recently the role of ABA catabolism in drought was supported by direct evidence from a wheat deletion line [63]. ABA is a major hot topic of drought research, and recently several studies are performed to determine its role in drought in relation to several drought-related molecules: protective proteins, NO, ROS, leaf and root growth, osmotic adjustment, BABA, ROS, and antioxidants [79, 82, 86, 95]. Additionally, in a recent study alginate oligosaccharides (AOS) prepared from degradation of alginate were shown to play a role in enhancing drought resistance in *T. aestivum* growth period by upregulating the drought tolerance related genes involved in ABA signal pathway, such as LEA1, SnRK2, and pyrroline-5-carboxylate synthetase gene (P5CS) [97].

### 5.3. Transgenic Studies for Identification of Gene Function

Transgenic plants provide the most straightforward way to demonstrate the functional relevance of the potential drought-related gene. Using functional genomics methods, modification of a single gene can be achieved in an identical genetic background. Analyzing the functional role of a protein of interest is achieved by the creation of overexpressor plants or loss-of-function mutants. These studies are most often carried out in model species, *Arabidopsis thaliana* or *Nicotiana benthamiana.* The advantages of these species are basically their rapid reproduction time and the ease of genetic transformation. However, other transgenic model systems, which hold the advantage of being phylogenetically more similar to monocot crop species, are also being developed, most importantly *Brachypodium distachyon*. The use of even a phylogenetically very close model plant in function verification of a gene of interest does not exclude the possibility that its role can differ in the crop of interest. Therefore transgenic studies applied directly on wheat are being developed but currently still more time and labor consuming. Other systems, which hold the advantage of straightforward analysis of gene function in the target crops are deletion lines and virus induced gene silencing (VIGS), which aids in functional characterization through silencing of targeted transcripts. 

#### 5.3.1. Overexpression Studies

Direct evidence for the functional role of several drought response candidates was established via their overexpression in *A. thaliana* or tobacco by *Agrobacterium* mediated transformation. To gain insight into the mechanisms the molecule of concern exerts a role in drought, and these studies are often coupled by the profiling of stress-related genes and characterization of the overexpressor plants in terms of their altered morphological and physiological properties. In recent years, drought-related molecular function of several transcription factors, signal transducers, and some other proteins were confirmed via their overexpression in *Arabidopsis* or *N. tabacum*. Information regarding these studies is summarized in Table 3. 

Recently, in four independent studies, overexpression of transcription factors (TaWRKY2, TaWRKY19, TaMYB33, TaPIMP1, and TaNAC) was shown to confer elevated drought tolerance in target model organisms [34, 37–39]. With the use of these overexpressor plants, WRKY proteins were shown to be involved in the DREB pathway [39], and MYB proteins were revealed to function in ROS detoxification [34, 38]. MYB TaPIMP1 overexpressors were also shown to exhibit increased levels of ABA synthesis and its restricted signaling [38]. Additionally, recently introgression of three kinases (TaABC1, TaSnRK2.7, and TaSnRK2.4) into *Arabidopsis* in separate studies was shown to improve drought tolerance evident by the water content and related measurements of overexpressor plants. All three kinases were observed to improve photosynthetic efficiency. TaABC1 was shown to be involved in DREB and ABA pathways [40]. TaSnRK2.7 was revealed to be involved in carbohydrate metabolism and mechanisms involving root growth [42]. TaSnRK2.4 overexpressors displayed differences in development and showed strengthened cell membrane stability [41]. In addition to transcription factors and kinases, overexpression studies of other proteins were also performed in model organisms, in relation to drought and drought-related capabilities. Cysteine protease, TaCP transgenics were observed to have higher survival rates under drought [45]. CHP rich zinc finger protein TaCHP was revealed to be involved in ABA-dependent and -independent signaling pathways [44]. Expansin, TaEXPR23, was shown to increase water retention ability and decrease osmotic potential [46]. Durum wheat aquaporins (TdPIP1; 1 and TdPIP2; 1) were revealed to regulate root and leaf growth [48]. Besides, in a recent study, *T. aestivum* salt induced protein (TaSIP) was shown to have a role in drought and salt tolerance via overexpression of the gene in *A. thaliana *resulted superior physiological properties [61] (Table 3).

#### 5.3.2. Wheat Deletion Lines and Virus Induced Gene Silencing

In recent years, a number of other transgenic studies were also performed for function determination. In such a study, *T. aestivum* deletion lines which lack ABA 8′-hydroxylase gene, involved in ABA catabolism, were used to study the role of ABA metabolism in the reproductive stage drought tolerance of cultivars. This study revealed a parallel between sensitivity to osmotic stress and higher spike ABA levels [63]. In a different study, ubiquitin:TaCHP transgenic wheat lines were used in studies in relation to the role of CHP rich zinc finger protein in drought stress [44]. Additionally, VIGS via barley stripe mosaic virus (BSMV) derived vectors was undertaken to silence ATG8 in wild emmer wheat, revealing it as a positive regulator of drought stress [33] (Table 3). VIGS was also used in another research in order to investigate the roles of *Era1 *(enhanced response to abscisic acid), *Sal1 *(inositol polyphosphate 1-phosphatase), and *Cyp707a* (ABA 8′-hydroxylase) in response to limiting water conditions in wheat. When subjected to limiting water conditions, VIGS-treated plants for *Era1* and *Sal1* resulted in increased relative water content (RWC), improved water use efficiency (WUE). Compared to other tested genes *Era1* was found to be the most promising as a potential target for drought tolerance breeding in wheat [49]. 

## 6. Improvement of Modern Wheat

Recent advances in molecular biological, functional, and comparative tools open up new opportunities for the molecular improvement of modern wheat. Recently developed techniques enable faster identification and characterization of drought-related gene(s) and gene region(s). Natural variants of modern species harbor a large repertoire of potential drought-related genes and hold a tremendous potential for wheat improvement. Introduction of drought-related components of wheat can be performed either with breeding through marker-assisted selection or transgenic methods. Recent increase in sequence availability due to recently developed high-throughput sequencing strategies has provided several high quality genetic markers for breeding. Transgenic strategies with enhanced transformation and selection methods are currently being developed.

### 6.1. Marker-Assisted Selection

Molecular breeding approaches based on specific traits utilize molecular markers for the screening of drought tolerance in cultivars. Loci that are targeted in marker-assisted selection (MAS) are most often derived from QTL mapping studies of quantitative traits [98]. MAS is most often performed based on physiomorphological characteristics related to yield under drought conditions. Markers that are utilized in such a context include SSR (simple sequence repeat) markers, *Xgwm136,* and NW3106, which are linked to genes that effect tillering capacity and coleoptile length, respectively [99]. Other selection markers are linked to Rht (reduced height) genes, which are known to be associated with harvest index. Additionally, transcription factor-derived markers, especially DREB proteins hold a great potential as PCR-based selection markers that can be useful in MAS [100]. However, the isolation of transcription factors is a challenge since they belong to large gene families containing members with high sequence similarities. Identification and successful isolation of a single drought-related loci is compelling also in general due to the complex genomic structure of wheat. However, in the near future, completion of wheat genome sequencing will pace identification of specific loci and the development of markers to be used in selection during breeding processes [98, 101]. 

### 6.2. Use of Transgenics

An alternative to ongoing breeding programmes is transgenic methods, which enable the transfer of only the desired loci from a source organism to elite wheat cultivars, avoiding possible decrease in yield due to the cotransfer of unwanted adjacent gene segments. Until now, transcription factors have been the most appealing targets for transgenic wheat improvement, due to their role in multiple stress-related pathways. In two different lines of research, overexpression of cotton and *A. thaliana *DREB was performed in wheat, resulting in transgenic lines with improved drought tolerance [102–104]. In another study, a barley LEA protein, *HVA1*, was also overexpressed in wheat, and overexpressors were observed to have better drought tolerance [105]. Transgenic wheat obtained with *Arabidopsis* DREB and *HVA1* protein overexpression was also shown to produce higher yield in the field under drought conditions, but further studies are required to confirm their performance under different environments [105]. 

It is not unreasonable to predict in the following decades: GM (genetically modified) wheat will be transferred to the fields as a common commercial crop. However, to pace this process, new transgenics methodologies should be developed since the current methods are laborious and time consuming. In a recent study, drought enhancement of bread wheat was established with the overexpression of barley *HVA1,* using a novel technique, which combines doubled haploid technology and *Agrobacterium* mediated genetic transformation [62] (Table 3).

### 6.3. Use of Proteomics

Despite the impressive technological breakthroughs in the genomics of drought resistant cultivars the overall scenario is not so promising, and new dimensions have to be explored for the exact elucidation of the wheat drought response process. Hence new studies are focusing to study wheat tolerance at the proteomic level to target different proteins and understand their role in stress. One particular study during grain development used comparative proteomic analysis and used 2 varieties of wheat resistant (CIMMYT wheat variety Kauz) and sensitive (Janz to drought). They applied linear and nonlinear 2-DE and MALDI-TOF mass spectrometry and elucidated that non-linear 2-DE showed a high resolution and identifies 153 spots of proteins that were differentially expressed, 122 of which were detected by MALDI-TOF. The characterized proteins were primarily metabolism proteins (26% carbohydrate metabolism), proteins involved in defense and detoxification (23%), and the rest of 17% were storage proteins. The study successfully showed the differential expression of various proteins in drought resistant and tolerant varieties. Kauz wheat variety showed high expression of LEA and alpha-amylase inhibitors and catalase isozyme 1, WD40 repeat protein, whereas these proteins were either unchanged or downregulated in Janz variety. Vice versa ascorbate peroxidase G beta-like protein and ADP glucose pyrophosphorylase remained unchanged in Kauz but were all downregulated in Janz. Proteins such as triticin precursor and sucrose synthase showed a considerably higher expression in Kauz water deficit variety compared to Janz water deficit plants. Thus the differential expression shows that biochemical and protein level expression could be a simpler approach to understanding and manipulating drought stress in plants [106].

A parallel approach to understanding the protein expression and posttranslational modification in wheat was carried out by Budak et al. in which 2 wild varieties of emmer wheat *Triticum turgidum* ssp. *dicoccoides *TR39477 and TTD22 were used along with one modern wheat cultivar *Triticum turgidum* ssp.* durum *cv. Kızıltan. The complete leaf proteome profiles of all three genotypes were compared by 2-DE gel electrophoresis and nanoscale liquid chromatographic electrospray ionization tandem mass spectrometry. Instead of using only drought tolerant and drought resistant varieties another third intermediate variety (modern) was also used. Although many proteins were common in all 3 cultivars both modern and durum but 75 differentially expressed proteins were detected [107]. Consequently comparative proteomics may provide a clearer picture and alternate way to evaluate and characterize drought resistant genes and proteins in wheat varieties.

## 7. Conclusion and Future Perspectives

Drought stress is one of the major limitations to crop production. To develop improved cultivars with enhanced tolerance to drought stress, identification of osmotic stress-related molecules and determination of their roles and locations in several physiological, biochemical, and gene regulatory networks is necessary. Several QTLs for key morphopysiological characteristics and yield were identified under water-limited conditions through creation of linkage maps using parentals with different drought coping abilities. In recent decades, application of high-throughput screening, “omics” strategies on *Triticum* species with differential drought tolerance coping abilities, has revealed several stress-related candidate gene(s) or gene block(s). Furthermore, using a variety of bioinformatics, molecular biology, and functional genomics tools, drought-related candidates were characterized, and their roles in drought tolerance were studied. Major drought-related molecules were revealed to be signal transduction pathway components and transcription factors. Several osmoprotectants, compatible solutes, ROS, and antioxidants were shown to accumulate in response to dehydration. Drought stress was found to alter various ongoing metabolic processes, such as growth, photosynthesis, and respiration. 

Analysis of drought response has been complicated in the absence of wheat genomic sequence data. However, with the recent advances in sequencing technologies, genome sequence of bread wheat is almost complete by the efforts of ITMI (The International *Triticeae *Mapping Initiative) and IWGSC (International Wheat Genome Sequencing Consortium). Availability of whole wheat genome sequence will contribute to the ongoing studies of exploring the extensive reservoir of alleles in drought tolerant wild germplasm, and this also enables better marker development, genome analysis and large scale profiling experiments. “Omics” strategies have especially contributed to drought research since osmotic stress response is not only genomic based but also regulated at the posttranscriptional and posttranslational levels. Advances in transformation/selection strategies have paced molecular transformation of wheat, which has an advantage to conventional and marker-assisted breeding for targeted introduction of only the desired loci. 

It is reasonable to predict that in the following years higher yielding wheat under drought conditions will be developed through breeding or molecular transformation of novel genes obtained from screening of wheat germplasms and will be commercially grown to balance the production with the consumption of the increasing human population. Research exploiting recent advances in genomics technologies has made it possible to dissect and resynthesize molecular regulation of drought and manipulate crop genomes for drought tolerance. The future efforts will be to integrate and translate these resources into practical higher yielding field products. 

## Supplementary Material

Table 1: This table lists all QTLs related to drought stress, identified in wheat, in the last three years.
Table 2: This table lists studies on drought related molecular mechanisms and molecules in wheat, in the last three years.

## Figures and Tables

**Table 1:** Transcript, protein, metabolite profiling studies conducted in the last three years.

Species	Cultivars	Tissue	Drought stress application	Method	Reference
*T. aestivum *	Drought tolerance: Plainsman V: tolerant; Kobomugi: sensitive	Root	Moderate drought stress applied on tillering stage	cDNA microarray	[7]
*T. aestivum *	Drought tolerance: information can not be accessed	Grain	Short water shortage in early grain development	cDNA microarray	[8]
*T. aestivum *	Efficiency of stem reserve mobilization in peduncles: N49: tolerant; N14: sensitive	Stem	Progressive drought stress after anthesis	2D gel and MS	[9]
*T. aestivum *	Cultivar Vinjett	Grain	Drought applied at terminal spiklet or at anthesis	2D gel and MS	[10]
*T. aestivum *	Yield under drought: Excalibur: tolerant; RAC875: tolerant; Kukri: sensitive	Leaf	Cyclic drought applied after first flag leaf formation mimicking field conditions	SCX column HPLC and MS	[11]
*T. durum *	Able to acquire drought tolerance: Ofanto: tolerant	Leaf	Drought applied at booting stage (controls SWC: irrigated when it decreases %50 of field capacity; drought SWC: irrigated when it decreases %12.5 of field capacity)	cDNA-AFLP	[12]
*T. durum *	Drought tolerance: Om Rabia3: tolerant; Mahmoudi: sensitive	Embryo	Drought applied at final development stage of seed maturity	2D gel and HPRP column and MS	[13]
*T. dicoccoides *	Yield under drought conditions: Y12-3: tolerant; A24-39: sensitive	Leaf	Terminal drought applied at inflorescence emergence stage	Transcript profiling	[14]
*T. dicoccoides *	Yield under drought conditions: Y12-3: tolerant; A24-39: sensitive	Leaf	Drought applied after germination at five/six leaf stage	Transcript and metabolite profiling	[14]
*T. dicoccoides *	Drought tolerance: TR39477: tolerant; TR38828: tolerant	Leaf/root	Shock drought stress	miRNA profiling	[15]

*T*: *Triticum*; SWC: soil water content; 2D: 2-dimensional; SCX: strong cation exchange; HPLC: high performance liquid chromatography; MS: mass spectrometry; cDNA: complementary DNA; AFLP: amplified fragment length polymorphisms; HPRP: human prion protein.

**Table 2:** List of identified and characterized drought related genes in the last three years.

Gene	Function	Related mechanism/stress	Reference
TaPIMP1	Transcription factor: R2R3 type MYB TF	Drought	[34]
TaSRG	Transcription factor: *Triticum aestivum *salt response gene	Drought	[35]
TaMYB3R1	Transcription factor: MYB3R type MYB TF	drought	[36]
TaNAC (NAM/ATAF/CUC)	Transcription factor: plant specific NAC (NAM/ATAF/CUC) TF	Drought	[37]
TaMYB33	Transcription factor: R2R3 type MYB TF	Drought	[38]
TaWRKY2, TaWRKY19	Transcription factor: WRKY type TF	Drought	[39]
TdicDRF1	Transcription factor: DRE binding protein	Drought	[32]
TaABC1	Kinase: protein kinase ABC1 (activity of bc(1) complex)	Drought	[40]
TaSnRK2.4	Kinase: SNF1 type serine/threonine protein kinase	Drought	[41]
TaSnRK2.7	Kinase: SNF1 type serine/threonine protein kinase	drought	[42]
TdTMKP1	Phosphatase: MAP kinase phosphatase	Drought	[43]
TaCHP	CHP rich zinc finger protein with unknown function	ABA-dependent and -independent pathways	[44]
TaCP	Protein degradation: cysteine protease	Drought	[45]
TaEXPR23	Cell wall expansion: expansin	Water retention ability and osmotic potential	[46]
TaL5	Nucleocytoplasmic transport of 5S ribosomal RNA: ribosomal L5 gene	Drought	[47]
TdPIP1;1, TdPIP1;2	Protective protein: aquaporin	Drought	[48]
TdicATG8	Autophagy: autophagy related gene 8	Drought	[33]
TdicTMPIT1	Autophagy: integral transmembrane protein inducible by TNF-*α*	Drought	[31]
*Era1*, *Sal1 *	Enhanced response to ABA, inositol polyphosphate 1-phosphatase	Drought	[49]

Ta: *Triticum aestivum*; Td: *Triticum durum*; Tdic: *Triticum dicoccoides*; DRE: drought related element; SNF: Sucrose nonfermenting; MAP: mitogen activated protein; ABA: abscisic acid; CHP: cysteine histidine proline; TNF-*α*: tumor necrosis factor *α*; PIMP: pathogen induced membrane protein; CP: cysteine protease; EXPR: expansin; PIP: plasma membrane intrinsic proteins.

**Table 3:** List of genes confirmed to function in drought by transgenic studies in last three years.

Type of transgenic study	Source of the gene	Gene	Function	Related mechanism/stress	Reference
Overexpression in *A. thaliana *	From *T. Aestivum *	WRKY2, WRKY19	Transcription factor: WRKY type TF	Drought	[39]
Overexpression in *A. thaliana *	From *T. Aestivum *	MYB33	transcription factor: R2R3 type MYB TF	Drought	[38]
Overexpression in *N. tabacum *	From *T. Aestivum *	PIMP1	Transcription factor: R2R3 type MYB TF	Drought	[34]
Overexpression in *N. tabacum *	From *T. Aestivum *	NAC (NAM/ATAF /CUC)	Transcription factor: plant-specific NAC (NAM/ATAF/CUC) TF	Drought	[37]
Overexpression in *A. thaliana *	From *T. Aestivum *	ABC1	Kinase: protein kinase ABC1 (activity of bc(1) complex)	Drought	[40]
Overexpression in *A. thaliana *	From *T. Aestivum *	SnRK2.4	Kinase: SNF1-type serine/threonine protein kinase	Drought	[41]
Overexpression in *A. thaliana *	From *T. Aestivum *	SnRK2.7	Kinase: SNF1-type serine/threonine protein kinase	Drought	[42]
Overexpression in *A. thaliana *	From *T. Aestivum *	CP	Protein degradation: cysteine protease	Drought	[45]
Overexpression in *A. thaliana *	From *T. Aestivum *	CHP	CHP rich zinc finger protein with unknown function	ABA-dependent and -independent pathways	[44]
Overexpression in *N. tabacum *	From *T. Aestivum *	EXPR23	Cell wall expansion: expansin	Water retention ability and osmotic potential	[46]
Overexpression in *A. thaliana *	From *T. Aestivum *	TaSIP	Salt induced protein with unknown function	Drought and salinity	[61]
Overexpression in *N. tabacum *	From *T. Durum *	PIP1;1, PIP1;2	Protective protein: aquaporin	Drought	[48]
Overexpression in *T. aestivum *	From *H. Vulgare *	HVAI	Protective protein: LEA	Drought	[62]
Transgenic ubiquitin: TaCHP	—	CHP	CHP rich zinc finger protein with unknown function	ABA-dependent and -independent pathways	[44]
TaABA08′OF1 deletion line	—	ABA08	ABA catabolism: ABA 8′-hydroxylase	Drought	[63]
VIGS silencing in *T*. *dicoccoides *	—	ATG8	Autophagy: autophagy related gene 8	Drought	[33]
VIGS silencing in *T. aestivum *	—	*Era1*, *Sal1 *	Enhanced response to ABA, inositol polyphosphate 1-phosphatase	Drought	[49]

ABA: abscisic acid; CHP: cysteine histidine proline; SNF: sucrose nonfermenting; PIMP: pathogen induced membrane protein; CP: cysteine protease; EXPR: expansin; PIP: plasma membrane intrinsic proteins; LEA: late embryogenesis abundant; HVA: *Hordeum vulgare* aleurone; TaSIP: *Triticum aestivum* salt induced protein; VIGS: virus induced gene silencing.
