# Supplementary material for: Drought Tolerance in Modern and Wild Wheat
Source: ScientificWorldJournal. 2013 May 15;2013:548246. doi: 10.1155/2013/548246 (PMC3671283; doi:10.1155/2013/548246)
Supplement: Supplementary file 1 — Table 1: This table lists all QTLs related to drought stress, identified in wheat, in the last three years. Table 2: This table lists studies on drought related molecular mechanisms and molecules in wheat, in the last three years. [file 548246.f1.doc]

| **Supplementary Table 1 Drought Related QTLs Identified in the Last Three Years** | | | |  |
| --- | --- | --- | --- | --- |
|  |  |  |  |  |
| **QTL** | **Trait** | **Chromosomal location** | **Drought condition** | **Reference** |
|  |  |  |  |  |
| *QEet.aww* | Days to ear emergence | 2D | BOL06 | Bennet et al, 2012a |
| *QEet.aww* | Days to ear emergence | 2B | BOL06 | Bennet et al, 2012a |
| *QEet.aww* | Days to ear emergence | 7A | BOL06 | Bennet et al, 2012a |
| *QEet.aww* | Days to ear emergence | 7A | BOL06 | Bennet et al, 2012a |
| *QEet.aww* | Days to ear emergence | 5B | BOL06 | Bennet et al, 2012a |
| *QEet.aww* | Days to ear emergence | 7B | BOL06 | Bennet et al, 2012a |
| *QEet.aww* | Days to ear emergence | 5A | BOL06 | Bennet et al, 2012a |
| *QEet.aww* | Days to ear emergence | 2D | MIN06 and BOL06 | Bennet et al, 2012a |
| *QEet.aww* | Days to ear emergence | 2B | MIN06 and BOL06 | Bennet et al, 2012a |
| *QEet.aww* | Days to ear emergence | 7A | MIN06 and BOL06 | Bennet et al, 2012a |
| *QEet.aww* | Days to ear emergence | 7A | MIN06 and BOL06 | Bennet et al, 2012a |
| *QEet.aww* | Days to ear emergence | 5A | MIN06 and BOL06 | Bennet et al, 2012a |
| *QEet.aww* | Days to ear emergence | 2D | CIMD07 | Bennet et al, 2012a |
| *QEet.aww* | Days to ear emergence | 2B | CIMD07 | Bennet et al, 2012a |
| *QZad.aww* | Zadoks score | 2D | BOL06 | Bennet et al, 2012a |
| *QZad.aww* | Zadoks score | 2B | BOL06 | Bennet et al, 2012a |
| *QZad.aww* | Zadoks score | 7A | BOL06 | Bennet et al, 2012a |
| *QZad.aww* | Zadoks score | 7B | BOL06 | Bennet et al, 2012a |
| *QZad.aww* | Zadoks score | 5A | BOL06 | Bennet et al, 2012a |
| *QZad.aww* | Zadoks score | 5B | BOL06 | Bennet et al, 2012a |
| *QZad.aww* | Zadoks score | 2D | MIN06 and BOL06 | Bennet et al, 2012a |
| *QZad.aww* | Zadoks score | 2B | MIN06 and BOL06 | Bennet et al, 2012a |
| *QZad.aww* | Zadoks score | 7A | MIN06 and BOL06 | Bennet et al, 2012a |
| *QZad.aww* | Zadoks score | 7A | MIN06 and BOL06 | Bennet et al, 2012a |
| *QW.aww* | Glaucousness | 2D | BOL06 | Bennet et al, 2012a |
| *QW.aww* | Glaucousness | 3A | BOL06 | Bennet et al, 2012a |
| *QW.aww* | Glaucousness | 6A | BOL06 | Bennet et al, 2012a |
| *QW.aww* | Glaucousness | 4D | BOL06 | Bennet et al, 2012a |
| *QW.aww* | Glaucousness | 3B | BOL06 | Bennet et al, 2012a |
| *QW.aww* | Glaucousness | 2D | MIN06 and BOL06 | Bennet et al, 2012a |
| *QW.aww* | Glaucousness | 2B | MIN06 and BOL06 | Bennet et al, 2012a |
| *QW.aww* | Glaucousness | 3D | MIN06 and BOL06 | Bennet et al, 2012a |
| *QW.aww* | Glaucousness | 3A | MIN06 and BOL06 | Bennet et al, 2012a |
| *QW.aww* | Glaucousness | 6A | MIN06 and BOL06 | Bennet et al, 2012a |
| *QW.aww* | Glaucousness | 3A | CIMD07 | Bennet et al, 2012a |
| *QW.aww* | Glaucousness | 2B | CIMD07 | Bennet et al, 2012a |
| *QW.aww* | Glaucousness | 3A | Glasshouse | Bennet et al, 2012a |
| QScr.aww | Drought | 1A | ENV1 | Bennet et al, 2012b |
| QKps.aww | Drought | 1A | ALL EXPTS | Bennet et al, 2012b |
| QKpsm.aww | Drought | 1A | ENV2 | Bennet et al, 2012b |
| QKpsm.aww | Drought | 1A | ENV1 | Bennet et al, 2012b |
| QYld.aww | Drought | 1A | ALL EXPTS | Bennet et al, 2012b |
| QKpsm.aww | Drought | 1A | ENV2-1 | Bennet et al, 2012b |
| QScr.aww | Drought | 1B | ENV3 | Bennet et al, 2012b |
| QScr.aww | Drought | 1B | ENV3-2 | Bennet et al, 2012b |
| QTwt.aww | Drought | 1B | ENV3-1 | Bennet et al, 2012b |
| QTwt.aww | Drought | 1B | ENV3 | Bennet et al, 2012b |
| QTwt.aww | Drought | 1B | ENV3-2 | Bennet et al, 2012b |
| QHi.aww | Drought | 1B | PID08 | Bennet et al, 2012b |
| QTn.aww | Drought | 1B | PID08 | Bennet et al, 2012b |
| QKpsl.aww | Drought | 1B | ENV2 | Bennet et al, 2012b |
| QKpsl.aww | Drought | 1B | ENV1 | Bennet et al, 2012b |
| QYld.aww | Drought | 1B | ENV2 | Bennet et al, 2012b |
| QYld.aww | Drought | 1B | ENV2-2 | Bennet et al, 2012b |
| QKps.aww | Drought | 1B | ALL EXPTS | Bennet et al, 2012b |
| QHt.aww | Drought | 1D | ALL EXPTS | Bennet et al, 2012b |
| QTkw.aww | Drought | 1D | ALL EXPTS | Bennet et al, 2012b |
| QTpa.aww | Drought | 1D | NUN08 | Bennet et al, 2012b |
| QTwt.aww | Drought | 1D | ENV3 | Bennet et al, 2012b |
| QScr.aww | Drought | 2A | ENV3-1 | Bennet et al, 2012b |
| QYld.aww | Drought | 2A | ENV3-1 | Bennet et al, 2012b |
| QTwt.aww | Drought | 2A | ENV2 | Bennet et al, 2012b |
| QTwt.aww | Drought | 2A | ENV3 | Bennet et al, 2012b |
| QTwt.aww | Drought | 2A | ENV3-1 | Bennet et al, 2012b |
| QHi.aww | Drought | 2A | ALL EXPTS | Bennet et al, 2012b |
| QEv.aww | Drought | 2B | ENV2 | Bennet et al, 2012b |
| QFll.aww | Drought | 2B | ALL EXPTS | Bennet et al, 2012b |
| QScr.aww | Drought | 2B | ENV3-2 | Bennet et al, 2012b |
| Qtpa.aww | Drought | 2B | MIN07 | Bennet et al, 2012b |
| QTkw.aww | Drought | 2B | ALL EXPTS | Bennet et al, 2012b |
| QFlw.aww | Drought | 2B | ALL EXPTS | Bennet et al, 2012b |
| QYld.aww | Drought | 2B | ENV2-2 | Bennet et al, 2012b |
| QYld.aww | Drought | 2B | ALL EXPTS | Bennet et al, 2012b |
| QTpa.aww | Drought | 2B | PID07 | Bennet et al, 2012b |
| QKpsm.aww | Drought | 2B | ENV3-1 | Bennet et al, 2012b |
| QScr.aww | Drought | 2B | ENV2-2 | Bennet et al, 2012b |
| QKpsm.aww | Drought | 2B | ENV3-2 | Bennet et al, 2012b |
| QKpsm.aww | Drought | 2B | ENV3 | Bennet et al, 2012b |
| QScr.aww | Drought | 2B | ENV3 | Bennet et al, 2012b |
| QScr.aww | Drought | 2B | ENV3-2 | Bennet et al, 2012b |
| QScr.aww | Drought | 2B | ENV2 | Bennet et al, 2012b |
| QScr.aww | Drought | 2B | ENV2-1 | Bennet et al, 2012b |
| QTkw.aww | Drought | 2B | ALL EXPTS | Bennet et al, 2012b |
| QTpa.aww | Drought | 2B | MIN07 | Bennet et al, 2012b |
| QKpsm.aww | Drought | 2D | ENV3-2 | Bennet et al, 2012b |
| QEn.aww | Drought | 2D | RAC08 | Bennet et al, 2012b |
| QFll.aww | Drought | 2D | ALL EXPTS | Bennet et al, 2012b |
| QEv.aww | Drought | 2D | ENV1 | Bennet et al, 2012b |
| QEv.aww | Drought | 2D | ENV3 | Bennet et al, 2012b |
| QEv.aww | Drought | 2D | ENV2 | Bennet et al, 2012b |
| QYld.aww | Drought | 2D | ENV3-1 | Bennet et al, 2012b |
| QHt.aww | Drought | 2D | ALL EXPTS | Bennet et al, 2012b |
| QFlw.aww | Drought | 2D | ALL EXPTS | Bennet et al, 2012b |
| QTpa.aww | Drought | 2D | NUN08 | Bennet et al, 2012b |
| QYld.aww | Drought | 2D | ENV1 | Bennet et al, 2012b |
| QKpsm.aww | Drought | 2D | ENV3-1 | Bennet et al, 2012b |
| QYld.aww | Drought | 2D | ENV3-1 | Bennet et al, 2012b |
| QHt.aww | Drought | 2D | ALL EXPTS | Bennet et al, 2012b |
| QKpsm.aww | Drought | 2D | ENV3 | Bennet et al, 2012b |
| QYld.aww | Drought | 2D | ENV2-1 | Bennet et al, 2012b |
| QYld.aww | Drought | 2D | ENV3 | Bennet et al, 2012b |
| QYld.aww | Drought | 2D | ENV3-2 | Bennet et al, 2012b |
| QYld.aww | Drought | 2D | ALL EXPTS | Bennet et al, 2012b |
| QYld.aww | Drought | 2D | ENV2 | Bennet et al, 2012b |
| QKpsm.aww | Drought | 2D | ENV2 | Bennet et al, 2012b |
| QWscc.aww | Drought | 2D | ALL EXPTS | Bennet et al, 2012b |
| QScr.aww | Drought | 2D | ENV2 | Bennet et al, 2012b |
| QKpsm.aww | Drought | 2D | ENV2-2 | Bennet et al, 2012b |
| QScr.aww | Drought | 2D | ENV2-2 | Bennet et al, 2012b |
| QPdl.aww | Drought | 3A | ALL EXPTS | Bennet et al, 2012b |
| QKps.aww | Drought | 3A | ALL EXPTS | Bennet et al, 2012b |
| QKpsm.aww | Drought | 3A | ENV1 | Bennet et al, 2012b |
| QTwt.aww | Drought | 3A | ENV3-2 | Bennet et al, 2012b |
| QHt.aww | Drought | 3A | ALL EXPTS | Bennet et al, 2012b |
| QTwt.aww | Drought | 3A | ENV2 | Bennet et al, 2012b |
| QWscc.aww | Drought | 3A | ALL EXPTS | Bennet et al, 2012b |
| QWsca.aww | Drought | 3A | RAC07wsca | Bennet et al, 2012b |
| QWsct.aww | Drought | 3A | RAC07wsct | Bennet et al, 2012b |
| QKpsm.aww | Drought | 3B | ENV2-1 | Bennet et al, 2012b |
| QScr.aww | Drought | 3B | ENV2 | Bennet et al, 2012b |
| QScr.aww | Drought | 3B | ENV2-1 | Bennet et al, 2012b |
| QPdl.aww | Drought | 3B | ALL EXPTS | Bennet et al, 2012b |
| QHi.aww | Drought | 3B | ALL EXPTS | Bennet et al, 2012b |
| QHi.aww | Drought | 3B | RAC07 | Bennet et al, 2012b |
| QEv.aww | Drought | 3B | ENV3 | Bennet et al, 2012b |
| QFll.aww | Drought | 3B | ALL EXPTS | Bennet et al, 2012b |
| QEv.aww | Drought | 3B | ENV1 | Bennet et al, 2012b |
| QScr.aww | Drought | 3B | ENV1 | Bennet et al, 2012b |
| QEv.aww | Drought | 3D | ENV3 | Bennet et al, 2012b |
| Qtn.aww | Drought | 3D | MIN07 | Bennet et al, 2012b |
| QBio.aww | Drought | 3D | ALL EXPTS | Bennet et al, 2012b |
| QHi.aww | Drought | 3D | ALL EXPTS | Bennet et al, 2012b |
| QTkw.aww | Drought | 3D | ALL EXPTS | Bennet et al, 2012b |
| QTwt.aww | Drought | 4A | ENV3 | Bennet et al, 2012b |
| QTn.aww | Drought | 4A | RAC08 | Bennet et al, 2012b |
| QTwt.aww | Drought | 4A | ENV3-1 | Bennet et al, 2012b |
| QFll.aww | Drought | 4A | ALL EXPTS | Bennet et al, 2012b |
| QTkw.aww | Drought | 4A | ALL EXPTS | Bennet et al, 2012b |
| QEn.aww | Drought | 4A | RAC08 | Bennet et al, 2012b |
| QKpsl.aww | Drought | 4B | ENV2 | Bennet et al, 2012b |
| QTpa.aww | Drought | 4B | PID07 | Bennet et al, 2012b |
| QScr.aww | Drought | 4D | ENV3-1 | Bennet et al, 2012b |
| QTwt.aww | Drought | 4D | ENV2 | Bennet et al, 2012b |
| QKpsm.aww | Drought | 4D | ENV2 | Bennet et al, 2012b |
| QKpsm.aww | Drought | 4D | ENV2-2 | Bennet et al, 2012b |
| QScr.aww | Drought | 4D | ENV3 | Bennet et al, 2012b |
| QScr.aww | Drought | 4D | ENV3-2 | Bennet et al, 2012b |
| QHi.aww | Drought | 4D | STR08 | Bennet et al, 2012b |
| QYld.aww | Drought | 4D | ENV2 | Bennet et al, 2012b |
| QYld.aww | Drought | 4D | ENV2-2 | Bennet et al, 2012b |
| QScr.aww | Drought | 5A | ENV3-1 | Bennet et al, 2012b |
| QFlw.aww | Drought | 5A | ALL EXPTS | Bennet et al, 2012b |
| QPdl.aww | Drought | 5A | ALL EXPTS | Bennet et al, 2012b |
| QHt.aww | Drought | 5A | ALL EXPTS | Bennet et al, 2012b |
| QScr.aww | Drought | 5B | ENV2-1 | Bennet et al, 2012b |
| QTkw.aww | Drought | 5B | ALL EXPTS | Bennet et al, 2012b |
| QFll.aww | Drought | 5B | ALL EXPTS | Bennet et al, 2012b |
| QEv.aww | Drought | 5B | ENV3 | Bennet et al, 2012b |
| QEv.aww | Drought | 6A | ENV1 | Bennet et al, 2012b |
| QKpsl.aww | Drought | 6A | ENV3 | Bennet et al, 2012b |
| QTwt.aww | Drought | 6A | ENV3 | Bennet et al, 2012b |
| QKpsl.aww | Drought | 6A | ENV1 | Bennet et al, 2012b |
| QKpsm.aww | Drought | 6A | ENV3 | Bennet et al, 2012b |
| QFlw.aww | Drought | 6A | ALL EXPTS | Bennet et al, 2012b |
| QScr.aww | Drought | 6A | ENV3-1 | Bennet et al, 2012b |
| QKpsm.aww | Drought | 6A | ENV3-1 | Bennet et al, 2012b |
| QTkw.aww | Drought | 6A | ALL EXPTS | Bennet et al, 2012b |
| Qtpa.aww | Drought | 6A | PID07 | Bennet et al, 2012b |
| QWsca.aww | Drought | 6A | RAC07wsca | Bennet et al, 2012b |
| QWsct.aww | Drought | 6A | RAC07wsct | Bennet et al, 2012b |
| QScr.aww | Drought | 6B | ENV3-1 | Bennet et al, 2012b |
| QTkw.aww | Drought | 6B | ALL EXPTS | Bennet et al, 2012b |
| QKpsm.aww | Drought | 6D | ENV3-2 | Bennet et al, 2012b |
| QYld.aww | Drought | 6D | ENV3-2 | Bennet et al, 2012b |
| QYld.aww | Drought | 6D | ENV2 | Bennet et al, 2012b |
| QKps.aww | Drought | 7A | ALL EXPTS | Bennet et al, 2012b |
| QKpsm.aww | Drought | 7A | ENV3-1 | Bennet et al, 2012b |
| QEv.aww | Drought | 7A | ENV2 | Bennet et al, 2012b |
| QYld.aww | Drought | 7A | ENV2 | Bennet et al, 2012b |
| QYld.aww | Drought | 7A | ALL EXPTS | Bennet et al, 2012b |
| QEv.aww | Drought | 7A | ENV1 | Bennet et al, 2012b |
| QYld.aww | Drought | 7A | ENV2-1 | Bennet et al, 2012b |
| QYld.aww | Drought | 7A | ENV3 | Bennet et al, 2012b |
| QYld.aww | Drought | 7A | ENV3-2 | Bennet et al, 2012b |
| QScr.aww | Drought | 7A | ENV3 | Bennet et al, 2012b |
| QScr.aww | Drought | 7A | ENV3-2 | Bennet et al, 2012b |
| QTkw.aww | Drought | 7A | ALL EXPTS | Bennet et al, 2012b |
| QYld.aww | Drought | 7A | ENV2-2 | Bennet et al, 2012b |
| QKpsm.aww | Drought | 7A | ENV3-2 | Bennet et al, 2012b |
| QHi.aww | Drought | 7A | STR08 | Bennet et al, 2012b |
| QKpsl.aww | Drought | 7A | ENV3 | Bennet et al, 2012b |
| QHi.aww | Drought | 7A | ALL EXPTS | Bennet et al, 2012b |
| QEv.aww | Drought | 7B | ENV1 | Bennet et al, 2012b |
| QKpsm.aww | Drought | 7B | ENV2-1 | Bennet et al, 2012b |
| QKpsm.aww | Drought | 7B | ENV3-2 | Bennet et al, 2012b |
| QFll.aww | Drought | 7B | ALL EXPTS | Bennet et al, 2012b |
| QTkw.aww | Drought | 7D | ALL EXPTS | Bennet et al, 2012b |
| QFll.aww | Drought | 7D | ALL EXPTS | Bennet et al, 2012b |
| Q.Spad.aww | Drought | 1A | Drt, Heat, Irr | Bennet et al, 2012c |
| Q.Phys.aww | Drought | 1B | Drt, Heat, Irr | Bennet et al, 2012c |
| Q.Ctgf.aww | Drought | 1B | Irr, Drt | Bennet et al, 2012c |
| Q.Ndvi.aww | Drought | 1B | Drt | Bennet et al, 2012c |
| Q.W.aww | Drought | 2A | Drt, Heat, Irr | Bennet et al, 2012c |
| Q.Phys.aww | Drought | 2A | Drt, Heat, Irr | Bennet et al, 2012c |
| Q.Ndvi.aww | Drought | 2A | Drt | Bennet et al, 2012c |
| Q.Eet.aww | Drought | 2B | Irr, Drt, Heat | Bennet et al, 2012c |
| Q.Tkw.aww | Drought | 2B | Irr, Drt, Heat | Bennet et al, 2012c |
| Q.Flw.aww | Drought | 2B | Heat, Drt | Bennet et al, 2012c |
| Q.Spad.aww | Drought | 2B | Drt | Bennet et al, 2012c |
| Q.W.aww | Drought | 2D | Drt, Heat, Irr | Bennet et al, 2012c |
| Q.Eet.aww | Drought | 2D | Irr, Drt, Heat | Bennet et al, 2012c |
| Q.Vig.aww | Drought | 2D | Heat, Drt | Bennet et al, 2012c |
| Q.Phys.aww | Drought | 2D | Drt, Heat, Irr | Bennet et al, 2012c |
| Q.Tkw.aww | Drought | 3A | Drt | Bennet et al, 2012c |
| Q.Yld.aww | Drought | 3A | Drt | Bennet et al, 2012c |
| Q.Pdl.aww | Drought | 3A | Drt, Heat, Irr | Bennet et al, 2012c |
| Q.W.aww | Drought | 3A | Drt, Heat, Irr | Bennet et al, 2012c |
| Q.Spad.aww | Drought | 3B | Drt, Heat, Irr | Bennet et al, 2012c |
| Q.Pdl.aww | Drought | 3B | Drt, Heat, Irr | Bennet et al, 2012c |
| Q.Yld.aww | Drought | 3B | Irr, Drt | Bennet et al, 2012c |
| Q.Kpsm.aww | Drought | 3D | Drt, Heat | Bennet et al, 2012c |
| Q.Yld.aww | Drought | 3D | Drt, Heat | Bennet et al, 2012c |
| Q.Vig.aww | Drought | 3D | Drt | Bennet et al, 2012c |
| Q.Ndvi.aww | Drought | 3D | Drt, Heat | Bennet et al, 2012c |
| Q.Ndvi.aww | Drought | 3D | Drt | Bennet et al, 2012c |
| Q.Ndvi.aww | Drought | 4A | Drt | Bennet et al, 2012c |
| Q.Vig.aww | Drought | 4A | Drt | Bennet et al, 2012c |
| Q.Spad.aww | Drought | 4B | Drt, Heat, Irr | Bennet et al, 2012c |
| Q.Pdl.aww | Drought | 4B | Drt, Heat, Irr | Bennet et al, 2012c |
| Q.Phys.aww | Drought | 4B | Drt, Heat, Irr | Bennet et al, 2012c |
| Q.Spad.aww | Drought | 4D | Drt, Heat, Irr | Bennet et al, 2012c |
| Q.Eet.aww | Drought | 5A | Drt, Heat | Bennet et al, 2012c |
| Q.Phys.aww | Drought | 5A | Drt, Heat, Irr | Bennet et al, 2012c |
| Q.Pdl.aww | Drought | 5A | Drt, Heat, Irr | Bennet et al, 2012c |
| Q.Vig.aww | Drought | 5B | Drt | Bennet et al, 2012c |
| Q.Ndvi.aww | Drought | 5B | Drt | Bennet et al, 2012c |
| Q.Tkw.aww | Drought | 5B | Drt | Bennet et al, 2012c |
| Q.Kpsm.aww | Drought | 5B | Drt | Bennet et al, 2012c |
| Q.Spad.aww | Drought | 5B | Drt, Heat, Irr | Bennet et al, 2012c |
| Q.Spad.aww | Drought | 6A | Irr, Drt | Bennet et al, 2012c |
| Q.Ctveg.aww | Drought | 6A | Irr | Bennet et al, 2012c |
| Q.Flw.aww | Drought | 6A | Irr | Bennet et al, 2012c |
| Q.Spad.aww | Drought | 6A | Drt, Heat, Irr | Bennet et al, 2012c |
| Q.Phys.aww | Drought | 6A | Drt, Heat, Irr | Bennet et al, 2012c |
| Q.Tkw.aww | Drought | 6A | Irr | Bennet et al, 2012c |
| Q.Ctgf.aww | Drought | 6B | Drt | Bennet et al, 2012c |
| Q.Spad.aww | Drought | 6B | Drt, Heat, Irr | Bennet et al, 2012c |
| Q.Spad.aww | Drought | 6B | Drt, Heat, Irr | Bennet et al, 2012c |
| Q.Tkw.aww | Drought | 2B | Drt | Bennet et al, 2012c |
| Q.Spad.aww | Drought | 6D | Drt, Heat, Irr | Bennet et al, 2012c |

Drt : sown under terminal drought with supplementary drip irrigation ; Heat : sown later than conventional practice to expose the plants to higher temperatures (2008, 2009), particularly during grain fill, but with supplementary irrigation to minimise the effect of water stress ; Irr : received full irrigation ; ENV1 : received a higher proportion of its growing season rainfall early in the vegetative growth stage, and was the warmest environment in most climatic variables; ENV2: was spread across the vegetative growth stage but like ENV1, had received around 95% of the seasons’ rainfall before reaching anthesis; ENV3: received slightly lower early season rainfall but during grain fill, still received approximately 20% of that environments’ rainfall; MIN07: Minnipa, SA (2007) 86 mm rainfall; NUN08: Nunjikompita, SA (2008) 96 mm rainfall; PID07: Piednippie, SA (2007) 113 mm rainfall, PID08: Piednippie, SA (2008) 212 mm rainfall; RAC07: Roseworthy, SA (2007) 153 mm rainfall; RAC08: Roseworthy, SA (2008) 223 mm rainfall; STR08: Streaky Bay, SA (2008) 95mm rainfallBOL06: (Booleroo) was selected as a site with a relatively cool winter and hot spring, with low rainfall ; MIN06 and BOL06: Minnipa (MIN06) and Booleroo are representative of a large proportion of the South Australian grain belt, frequently experiencing strong cyclical and terminal droughts; CIMD07: performance under water limitation
