# Supplementary material for: Drought Tolerance in Modern and Wild Wheat
Source: ScientificWorldJournal. 2013 May 15;2013:548246. doi: 10.1155/2013/548246 (PMC3671283; doi:10.1155/2013/548246)
Supplement: Supplementary file 2 [file 548246.f2.doc]

| **Supplementary Table II All Articles Published in Last Three Years, Classified in Relation to Drought Related Molecular Mechanism and Molecules** | | | | |  |
| --- | --- | --- | --- | --- | --- |
|  |  |  |  |  |  |
| **Drought Related Molecules and Pathway** | **Method** | **Species** | **Tissue** | **Related Mechanism/Stress** | **Reference** |
|  |  |  |  |  |  |
| **Compatible Solutes** |  |  |  |  |  |
| compatible solutes in response to SA and in relation to water status | biomass, relative leaf water content, photosynthetic pigments, inorganic, organic substance level measurements | T. aestivum | roots/shoots | drought | Loutfy et al, 2012 |
| compatible solutes in relation to osmotic adjustment | soil water content, water use (water content, water potential, relative water content, osmotic potential), growth (accumulation of dry matter), compatible solute measurements | T. aestivum | leaf | drought | Nio et al, 2011 |
| **Protective Proteins** |  |  |  |  |  |
| LEA, with a focus on dehydrins, in relation to ABA | transcript and protein level measurements and ABA quantification | T. aestivum | leaf | drought | Ji et al, 2011 |
| Aquaporins, Plasma membrane intrinsic proteins (PIPs) : TdPIP1;1, TdPIP1;2 | transgenics : ovexpression of TdPIP1;1, TdPIP1;2 in Nicotiana tabacum | T. durum | _ | drought | Ayadi et al, 2011 |
| Na+/K+ transporters : HKT-1 ( High affinity K+ transporter) | SNP and InDEL detection | T. durum | can not be accessed | drought | Mondini et al, 2012 |
| **Signalling** |  |  |  |  |  |
| Calcium sensor family kinases, Calcium dependent protein kinases (CDPKs) : TaCPK7, TaCPK12 | transcript level measurements | T. aestivum | leaf | ABA and PEG | Geng et al, 2011 |
| Sucrose nonferementing 1 (SNF1) type serine/threonine protein kinase (SnRK) : TaSnRK2.4 | transgenics : overexpression of TaSnRK2.4 in Arabidopsis thaliana | T. aestivum | _ | drought | Mao et al, 2010 |
| Sucrose nonferementing 1 (SNF1) type serine/threonine protein kinase (SnRK) : TaSnRK2.7 | transgenics : overexpression ofTaSnRK2.7 in Arabidopsis thaliana | T. aestivum | _ | drought | Zhang et al, 2011 |
| Mitogen activated protein (MAP) kinase phosphatase : TMKP1 | identification & characterization of TMKP1 | T. durum | _ | drought | Zaidi et al, 2010 |
| Protein kinase ABC1 (activity of bc(1) complex) : TaABC1 | transgenics : overexpression of TaABC1 in Arabidopsis thaliana | T. aestivum | _ | drought | Wang et al, 2011 |
| SA effect on compatible solutes and water status | biomass, relative leaf water content, photosynthetic pigments, inorganic, organic substance level measurements | T. aestivum | roots/shoots | drought | Loutfy et al, 2012 |
| NO effect on root growth; in relation to ABA and ROS | NO, ABA, ROS, root growth measurements | T. aestivum | root | drought | Tari et al, 2010 |
| **Photosynthesis and Respiration** |  |  |  |  |  |
| photosynthesis (PS II) in relation to ROS (CAT, APX, GR, SOD) | relative leaf water content, chlorophyll content, photosynthetic efficiency of PSII, protein content and enzyme activity measurements | T. durum/T. aestivum | leaf | drought | Huseynova et al, 2012 |
| heterogeneity of PSII ( reducing side heterogeneity and antenna heterogeneity) | reducing side heterogeneity and antenna heterogeneity measurements | T. aestivum | leaf | osmotic stress | Singh-Tomar et al, 2012 |
| photosynthesis | leaf chlorophyll content, maximum photochemical efficiency of PSII, stomatal conductance, yield measurements | T. aestivum | leaf | drought | Khamssi, et al, 2012 |
| gas exchange including photosynthesis and respiration | leaf water potential, respiration, gas exchange (stomatal conductance, transpiration, photosynthetic capacity) measurements | T. aestivum | leaf | drought | Vassileva et al, 2011 |
| **Growth** |  |  |  |  |  |
| Root growth : root elongation and root cell formation | observation of root with fluorescence microscopy | T. aestivum | root | osmotic stress |  |
| Root growth in relation to ROS, antioxidants with a focus on peroxidases | growth, relative water content, hydrogen peroxide level, transcript, protein, enzyme level measurements | T. aestivum | root | drought | Tari et al, 2010 |
| Root growth in response to NO ; in relation to ABA and ROS | NO, ABA, ROS, root growth measurements | T. aestivum | root | drought | Tari et al, 2010 |
| Leaf growth recovery : the role of ABA through osmotic adjustment | leaf elongation kinetics, water potential, osmotic potential, turgor pressure measurements | T. durum | leaf | drought | Mahdid et al, 2011 |
| expansin : TaEXPR23 | transgenics : overexpression of TaEXPR23 in Nicotiana tabacum | T. aestivum | _ | water retention ability and osmotic potential | Vassileva et al, 2011 |
| **Transcription Factors** |  |  |  |  |  |
| Dehydration responsive element binding (DREB) proteins : DREB1 ; WRKY- type transcription factors : WRK1 | SNP and InDELdetection | T. durum | can not be accessed | drought | Mondini et al, 2012 |
| MYB transcription factors : TaMYBsdu1 | transcript level measurements | T. aestivum | leaf/root | drought | Rahaie et al, 2010 |
| WRKY type transcription factors : TaWRKY2, TaWRKY19 | transgenics : overexpression of TaWRKY2, TaWRKY19 in Arabidopsis thaliana | T. aestivum | _ | drought | Niu et al, 2012 |
| R2R3 type MYB transcription factor : TaMYB33 | transgenics : overexpression of TaMYB33 in Arabidopsis thaliana | T. aestivum | _ | drought | Qin et al, 2012 |
| MYB3R type MYB transcription factor : TaMYB3R1 | identification & characterization of TaMYB3R1 | T. aestivum | _ | drought | Cai et al, 2011 |
| R2R3 type MYB transcription factor : Pathogen Induced Membrane Protein (PIMP) : TaPIMP1 | transgenics : overexpression of TaPIMP1 in Nicotiana tabacum | T. aestivum | _ | drought | Liu et al, 2011 |
| Plant specific NAC (NAM/ATAF/CUC) transcription factors : TaNAC2a | transgenics : overexpression of TaNAC2a in Nicotiana tabacum | T. aestivum | _ | drought | Tang et al, 2012 |
| Drought Responsive Element (DRE) binding protein : TdicDRF1 | identification & characterization of TdicDRF1 | T. dicoccoides | _ | drought | Lucas et al, 2011b |
| Triticum aestivum salt response gene : TaSRG | identification & characterization of TaSRG | T. aestivum | _ | drought | He et al, 2011 |
| **Post transcriptional/translational modifications** |  |  |  |  |  |
| miRNAs | miRNA profiling | dicoccoides | leaf/root | drought | Kantar, 2011 |
| proteolytic activity and cysteine proteases | leaf water status, pigment level, transcript, protein, enzyme activity level measurements | T. aestivum | leaf | drought | Simova-Stoilova et al, 2010 |
| cysteine protease : TaCP | transgenics : overexpression of TaCP in Arabidopsis thaliana | T. aestivum | _ | drought | Zang et al, 2010 |
| **ROS & Antioxidants** |  |  |  |  |  |
| ROS and antioxidants | hydrogen peroxidase, lipid peroxidation, glutathione/ascorbate level, enzyme activity measurements | T. aestivum | root cells/root mitochondria | drought | Selote et al, 2010 |
| antioxidants with a focus on ascorbate/glutathione cycle | trancript level measurements in stem ; ascorbate level, ascorbate peroxidase activity, lipid peroxidation measurements in leaf | T. aestivum | stem/leaf | drought | Secenji et al, 2010b |
| ROS (CAT, APX, GR, SOD) in relation to photosynthesis (PS II) | leaf relative water content, chlorophyll content, photosynthetic efficiency of PSII, protein content and enzyme activity measurements | T. durum/T. aestivum | leaf | drought | Huseynova et al, 2012 |
| ROS levels in response to NO ; in relation to ABA and root growth | NO, ABA, ROS, root growth measurements | T. aestivum | root | drought | Tari et al, 2010 |
| ROS levels in response to BABA; in relation to ABA | soil water content, dessiccation tolerance (relative leaf water content, lethal leaf water potential), ROS level, enzyme activity, lipid peroxidation, yield measurements, ABA quantification | T. aestivum | leaf | drought | Du et al, 2012 |
| **Abscisic Acid (ABA)** |  |  |  |  |  |
| ABA catabolism, ABA 8'-hydroxylase | transgenics : TaABA08'OF1 (ABA 8'-hydroxylase) deletion line | T. aestivum | _ | drought | Ji et al, 2011 |
| ABA in relation to protective proteins, LEA with a focus on dehydrins | transcript and protein level measurements and ABA quantification | T. aestivum | leaf | drought | Vaseva et al, 2010 |
| ABA in response to NO; in relation to ROS and root growth | NO, ABA, ROS, root growth measurements | T. aestivum | root | drought | Tari et al, 2010 |
| ABA in relation to recovery of leaf growth through osmotic adjustment | leaf elongation kinetics, water potential, osmotic potential, turgor pressure measurements | T. durum | leaf | drought | Mahdid et al, 2011 |
| ABA levels in response to BABA; in relation to ROS and antioxidants | soil water content, dessiccation tolerance (leaf relative water content, lethal leaf water potential), ROS level, enzyme activity, lipid peroxidation, yield measurements and ABA quantification | T. aestivum | leaf | drought | Du et al, 2012 |
| **Others** |  |  |  |  |  |
| BABA effect in relation to ABA, ROS and antioxidants | soil water content, dessiccation tolerance (leaf relative water content, lethal leaf water potential), ROS level, enzyme activity, lipid peroxidation, yield measurements and ABA quantification | T. aestivum | leaf | drought | Du et al, 2012 |
| polyamines : spermine, cadverine, spermidine | transcript level measurements | T. aestivum | shoot | drought | Kovács et al, 2010 |
| CHP rich zinc finger protein with unknown function : TaCHP | transgenics : Ubiquitin::TaCHP transgenic wheat lines , overexpression of TaCHP in Arabidopsis thaliana | T. aestivum | _ | ABA dependent and independent pathways | Li et al, 2010 |
| autophagy related gene 8 : TdicATG8 | transgenics : silencing in Triticum dicoccoides via VIGS | T. dicoccoides | _ | drought | Kuzuoglu-Ozturk, 2012 |
| ribosomal L5 gene : TaL5 | identification & characterization of TaL5 | T. aestivum | _ | drought | Kang et al, 2012 |
| integral transmembrane protein inducible by TNF-α (TMPIT) : TdicTMPIT1 | identification & characterization of TdicTMPIT1 | T. dicoccoides | _ | drought | Lucas et al, 2011a |

SA : Salicylic acid ; LEA : Late embryogenesis abundant ; ABA : Abscisic acid ; PSII : Photosystem II ; ROS : Reactive oxygen species ; NO : Nitric oxide ; SNP : Small Nucleotide Polymorphism ; InDEL : insertion/deletion ; VIGS : Virus induced gene silencing ; CAT : Catalase ; GR : Glutathione reductase ; SOD : Superoxide dismutase ; APX : Ascorbate peroxidase ; BABA : β-aminobutyric acid ; TNF-α : Tumor necrosis factor α ; CHP : Cysteine Histidine Proline
